# *Candidozyma auris* prevention practices in the United States: insights from the SHEA Research Network

**DOI:** 10.1017/ice.2026.10396

**Published:** 2026-04

**Authors:** Hannakate Lichota, McKenzi King, Rachel Medernach, Lahari Thotapalli, Ronda Cochran, Mary K. Hayden, Sarah E. Sansom

**Affiliations:** 1 Rush University Medical Center, Chicago, IL, USA; 2 The Consulting Division of Chestatee Pathology Associates, PC, Atlanta, GA, USA

## Abstract

**Objective:**

Understand current *Candidozyma auris* prevention practices in the United States and identify opportunities to improve containment.

**Design:**

Electronic survey.

**Setting:**

Acute care hospitals.

**Participants:**

Society for Healthcare Epidemiology (SHEA) Research Network (SRN) facilities located in the United States.

**Methods:**

REDCap survey distributed via email exploring knowledge and perceptions related to *C. auris* screening methods, prevention practices, barriers to prevention, and tools needed to improve containment.

**Results:**

Responses were received from 51/96 (53%) U.S.-based SRN facilities, with 80% identifying as teaching hospitals. Two-thirds of facilities (34/51) reported first-hand experience with *C. auris*, with 15/34 also experiencing at least one *C. auris* outbreak. Routine *C. auris* screening occurred in 47% (24/51) of facilities. *C. auris* prevention practices commonly included patient isolation, signage to notify staff of isolation status, and placement in a single patient room. When asked to identify barriers to control of *C. auris* at their facility, participants ranked lack of communication between healthcare facilities, lack of infection control at outside healthcare facilities, and lack of training as the top three barriers. *C. auris* prevention resources or tools perceived to be most helpful in their facility included effective decolonization regimens, standardized protocols for *C. auris* screening, and improved communication between healthcare facilities.

**Conclusion:**

SRN facilities commonly used isolation practices to prevent the spread of *C. auris*. Development of additional tools to improve prevention practices should target effective decolonization strategies and standardized screening protocols to support *C. auris* containment.

## Introduction

*Candidozyma auris* (*C. auris*) has emerged worldwide as a major healthcare-associated pathogen. *C. auris* causes serious invasive infections, rapidly contaminates the healthcare environment, and commonly demonstrates resistance to antifungal medications.^1–5^ *C. auris* has been implicated in outbreaks at many types of healthcare facilities, including short-term and long-term acute care hospitals (STACHS and LTACHs, respectively) and lower acuity skilled nursing facilities (SNFs).^6–12^

Within the United States, healthcare facilities such as acute care hospitals frequently rely on guidance from the U.S. Centers for Disease Control and Prevention (CDC) to inform their approach and prevent spread of healthcare associated pathogens.^13^ Current published CDC guidance for *C. auris* requires some interpretation by each facility based on the local *C. auris* epidemiology. Implementation of this guidance may vary between hospitals, potentially indicating a lack of knowledge or an inability to implement this guidance for financial or structural reasons. These limitations and knowledge gaps may create variability in infection prevention practices and limit containment.

The Society for Healthcare Epidemiology (SHEA) Research Network (SRN) is a group of healthcare facilities that collaborates to perform research on healthcare epidemiology and antimicrobial stewardship within the United States. We surveyed U.S.-based SRN acute care hospitals to better understand their approach to *C. auris* prevention practices, delineate perceived barriers to control, and to identify opportunities to improve containment.

## Methods

Our team developed a survey to assess facility characteristics, prevention practices, and barriers to *C. auris* prevention among acute care hospitals in the United States that participate in the SRN. This study was reviewed and approved by the Rush Institutional Review Board and by the SRN prior to email distribution using Research Electronic Data Capture (REDCap).^14^ The survey was distributed in 2024; result analysis was performed in 2025. To maximize response potential, surveys were sent out on a rolling basis with initial invitations extended, followed by three reminder emails. The survey consisted of 21 questions, including single or multiple-choice, ranked, and free text questions (Supplemental Methods). The total number of questions answered by each facility varied, with branching logic utilized to further clarify responses. Completion of all survey questions was not required for form submission, and facilities had the option to leave questions blank. Percentages were calculated against the denominator of the total pool of survey participants (*n* = 51) unless otherwise noted. This was done as total responses to questions varied and not all participants had the opportunity to view every question due to the nature of the branching logic employed. For ranked-choice questions, responses were analyzed as non-weighted counts. Responses were analyzed using descriptive statistics by SAS version 9.4 (Statistical Analysis System).^15^ When appropriate, qualitative responses were analyzed using an iterative process. Study team members (HL, SS) reviewed each free text response and qualitatively coded it by consensus.^16^

## Results

**Figure 1.**
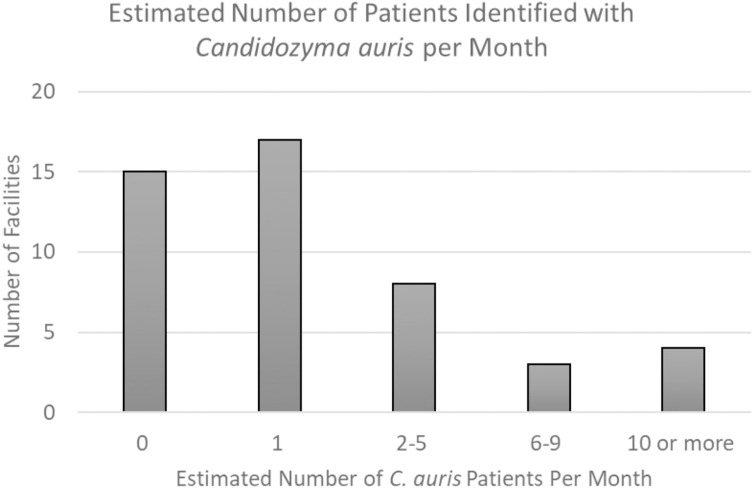
Estimated number of patients identified with *Candidozyma auris* per month, by facility.

**Figure 2.**
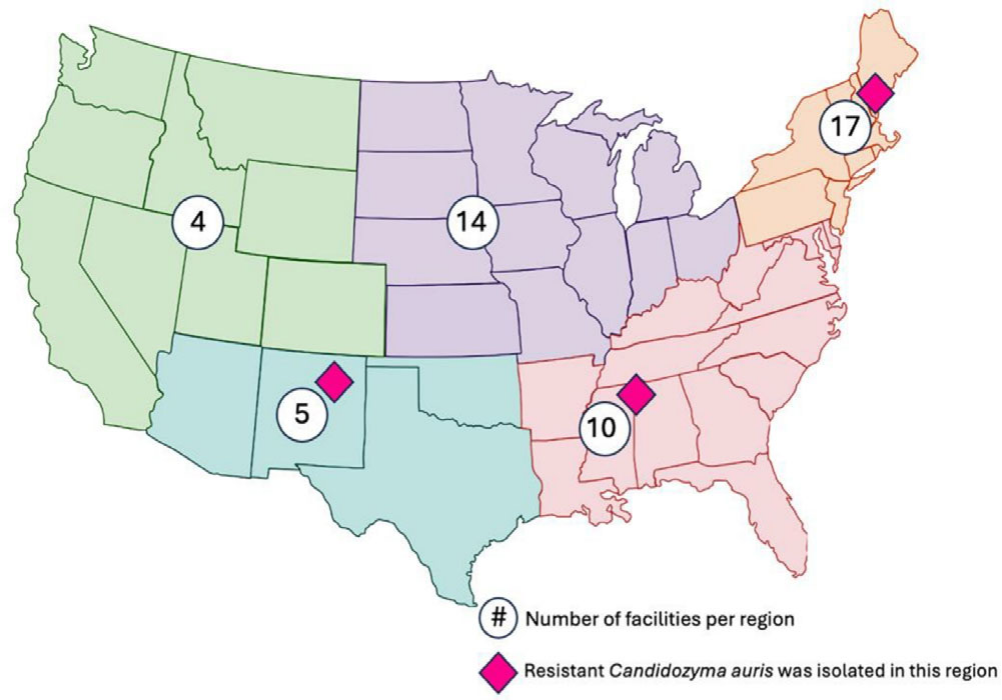
Locations of participating SHEA Research Network facilities and reported *C. auris* resistance. The number of participating acute care hospitals within each geographic region are shown in blue circles. Geographic regions include northeast, southeast, midwest, northwest, and southwestern United States. Pink diamonds indicate regions with multidrug-resistant *C. auris* (resistance ≥3 antifungal classes) reported by survey participants.

Among 96 acute care U.S.-based hospitals in the SRN, 51 responded (53% response rate) (Table 1). Thirty-four (67%) reported first-hand experience with *C. auris,* defined as having identified a patient infected or colonized with *C. auris* within their facility; half of these thirty-four facilities averaged at least one such patient per month (17/34, 50%) (Figure 1). Fifteen hospitals also reported experience with at least one *C. auris* outbreak, defined as suspected transmission of *C. auris* within their facility. Ten percent of hospitals (5/51) reported identification of a *C. auris* isolate with resistance to ≥3 antifungal medication classes (Figure 2).

**Table 1.** Characteristics of participating SRN facilities

Facility characteristics	Number of facilities (N = 51), (%)
**Self-identified facility type** ^**a**^	
Teaching hospital	41 (80)
Academic hospital	11 (22)
Veterans Affairs hospital	8 (16)
Community hospital	7 (14)
Tertiary care hospital	6 (12)
Private hospital	3 (6)
**Bed capacity of each facility**	
>500 beds	32 (62)
200–500 beds	9 (18)
<200 beds	9 (18)
Did not respond	1 (2)
**Most common room type(s)** ^**a**^	
Single patient rooms	37 (73)
Shared rooms (≥2 occupants).	1 (2)
Did not respond	13 (25)
**Infection control committee membership** ^**a**^	
Nurse, infection control certified	47 (92)
Hospital epidemiologist	46 (90)
Infectious disease physician	43 (84)
Microbiologist	26 (51)
Employee/occupational health staff	24 (47)
Environmental services staff	22 (43)
Nurse, not infection control certified	19 (37)
Hospital Administrator	19 (37)
Pharmacist	17 (33)
Other physician specialty^b^	10 (20)
Advanced practice providers and/or nurse practitioners	5 (10)

Note. ^a^Categories not mutually exclusive.

b Other physician specialties included by free text: critical care (5), general surgery or surgical subspecialties (4), hospitalist (3), pediatrics (2), anesthesiology (2), cardiology (1), oncology (1).

*C. auris* screening was performed in 24 (47%) acute care hospitals, typically at the time of admission (22/24, 92%). Facilities were asked to describe only their own screening practices, excluding ongoing public health screening. Sixteen facilities screened by two body sites (axillae and groin), while seven screened by three body sites (axillae, groin, and anterior nares). Polymerase chain reaction was the most used testing method (17/24, 71%). Acute care hospitals using targeted *C. auris* screening strategies (14/24, 58%) were asked to share screening criteria in a free text response; approaches included screening patients who were exposed to other healthcare facilities (eg, SNF, LTACH) (*n* = 12), history of a multidrug-resistant organism (*n* = 7), healthcare exposure outside of the U.S. (*n* = 6), presence of a tracheostomy or chronic ventilation (*n* = 5), admission from a facility with a known *C. auris* outbreak (*n* = 5), or presence of a chronic wound (*n* = 1).

Thirty-four acute care hospitals (67%) reported having a written policy regarding *C. auris* prevention; four respondents declined to answer this question. Isolation was commonly reported as a prevention practice for patients with *C. auris*, including contact isolation (ie, use of gloves and gowns) (88%), placement into a single patient room (86%), use of signage to notify staff of isolation status (86%), and preemptive isolation while awaiting screening results (45%). Most hospitals reported the use of cleaning products on the Environmental Protection Agency (EPA) list P for *C. auris* disinfection (71%).^17^ Targeted prevention practices related to environmental cleaning were reported in less than half of participants (Table 2).

**Table 2.** Infection prevention and control practices for *Candidozyma auris*

Infection prevention and control practices	Number of facilities^a^
**Screening practices**	**(N = 51), (%)**
Performs any screening for *C. auris* ^b^	24 (47)
Among facilities who endorse performing screening for *C. auris:*	**(N = 24), (%)**
Performs screening at the time of hospital admission Preemptive isolation while awaiting result of *C. auris* screening test result	22 (92) 23 (96)
Body sites targeted for screening Two body sites (axilla, groin) Three body sites (axilla, groin, anterior nares)	16 (66) 7 (29)
**Isolation practices**	**(N = 51), (%)**
Contact isolation (use of gown and gloves)	45 (88)
Signage at patient room to notify staff of isolation status	44 (86)
Placement in a single patient room	44 (86)
**Environmental disinfection practices**	**(N = 51), (%)**
Ensure use of cleaning products with claims for *C. auris* (EPA List P)^17^	36 (71)
Enhanced cleaning of shared patient equipment	24 (47)
Increased frequency of environmental disinfection of patient rooms	19 (37)
**Other practices**	**(N = 51), (%)**
Facility has written policy on *C. auris* infection prevention and control^b^	34 (66)
Antiseptic bathing of patients	14 (27)
Cohorting of patients (ie, shared space with other patients who have *C. auris*)	8 (16)
Cohorting of healthcare staff to care for patients with *C. auris*	6 (12)

Note. ^a^Respondents were not required to complete every question, and percentages are calculated against total pool of respondents (N = 51) unless otherwise specified.

b Four respondents declined to answer this question.

Barriers to control of *C. auris* were queried as a ranked choice question; the three most commonly selected barriers included lack of communication of *C. auris* colonization or infection status at the time of patient transfer between healthcare facilities (*n* = 32), lack of infection control at outside healthcare facilities (*n* = 25), and lack of training for frontline staff (*n* = 21) (Table 3). Participants were also asked to rank which resources or tools would best support *C. auris* prevention in their hospital; the three most commonly selected tools were *C. auris* decolonization regimens (*n* = 28), standardized protocols for *C. auris* screening (*n* = 22), and improved communication between healthcare facilities (*n* = 20) (Table 4).

**Table 3.** In your opinion, what are the most important barriers that should be addressed to prevent the spread of *C. auris* in your facility?

Barrier Ranked in Top 3 of Importance	N
Lack of communication about patient *C. auris* status between healthcare facilities	32
Lack of infection control at outside healthcare facilities	25
Lack of training for frontline staff	21
Lack of electronic health record integration across facilities	18
Lack of microbiologic and/or diagnostic services	10
Other^a^	8
Lack of access to information about infections	6
Space constraints (inability to isolate in single room)	6
Lack of staffing	5
Lack of administrative support	3
Lack of access to information on infection control	1

Note. ^a^Free text responses included public health guidance or risk stratification tools to select patients for screening (n = 4); communication with public health departments (e, lack of data sharing, delay in obtaining genomic data) (n = 3); non-adherence with infection control measures (n = 2); lack of availability of microbiologic resources to support screening (n = 1).

**Table 4.** In your opinion, development of which of the following tools would be most helpful to support your ability to respond to *C. auris* in your facility?

Helpful Tools Ranked in Top 3 of Importance	N
Effective patient decolonization regimen for *C. auris*	28
Standardized protocols for *C. auris* screening	22
Improved communication at time of transfer of *C. auris*-positive patients between facilities	20
New or improved environmental disinfection protocols	16
Faster turnaround time for *C. auris* screening tests	15
New or improved environmental disinfection tools	11
Improved training resources for frontline staff	8
Standardized protocols for *C. auris* isolation	3
Standardized protocols for *C. auris* treatment	2
Improved training resources for infection prevention staff	1
Other^a^	1

Note. ^a^Free text response: coordinated regional surveillance program.

## Discussion

*Candidozyma auris* continues to spread across U.S. healthcare facilities despite containment efforts.^7^ We surveyed *C. auris* prevention practices in U.S.-based acute care SRN hospitals to better understand barriers to control and identify opportunities to improve prevention strategies. Two-thirds of participating hospitals reported having provided care to a patient with *C. auris* colonization or infection, with half of those also experiencing at least one *C. auris* outbreak. Prevention strategies commonly included contact isolation, with approximately half of hospitals surveyed also performing *C. auris* screening. Barriers to effective *C. auris* prevention focused on challenges when accepting patients from other healthcare facilities, including poor communication regarding *C. auris* status and perceived suboptimal infection control practices at the originating healthcare facility. The most helpful tools to improve *C. auris* containment included the development of effective decolonization regimens and standardized screening strategies.

Reported *C. auris* prevention practices varied among participating facilities, and there was no single element that was universally applied across SRN acute care hospitals. There was strong, but not universal, alignment across facilities regarding use of contact isolation, placement into single occupant rooms, and use of signage to notify staff of isolation status. Future prevention efforts should focus on aligning these critical prevention practices, as *C. auris* is known to rapidly contaminate the healthcare environment and may serve as an important intermediary of transmission.^18^ In contrast to other prevention practices, the use of targeted environmental disinfection was less consistent, although over 70% of participants did report ensuring the use of EPA list P products for environmental disinfection.^17^ Survey questions were not tailored to explore the reasoning behind facilities’ use of disinfectants outside of EPA list P products, and this represents an area for future research. Our current findings suggest that additional education and training in environmental disinfection is an area of opportunity to prevent *C. auris* spread, potentially through multimodal training approaches (ie, in-person teaching by experts as well as self-guided modules).^19^

Our study adds to the growing body of literature regarding screening practices for *C. auris*. In a survey of 253 Emerging Infection Network facilities, 36% reported screening patients for *C. auris*.^20^ Another survey of French mycologists from facilities in France (*n* = 36) found that 31% were aware of *C. auris* screening being conducted in their facility.^21^ In the current survey, nearly 50% of participants reported screening for *C. auris*, which may reflect growing awareness of *C. auris* or the greater resources available to teaching hospitals,^22^ who comprised most survey participants. Barriers to screening for *C. auris* in this study aligned with prior work,^20,21^ including limited microbiologic laboratory capabilities. Participants noted the development of standardized screening protocols for *C. auris* as the second most commonly reported tool to help improve *C. auris* containment. Expanded engagement of local public health departments to provide guidance and microbiologic support for screening may help contain *C. auris* spread. Additionally, development of strong regional collaborations between healthcare facilities to contain *C. auris* may be needed, which would facilitate alignment of screening protocols and communication of screening results.

Effective communication of patient *C. auris* status between healthcare facilities at the time of patient transfer was identified as an important barrier across multiple questions in our survey. Interfacility transfer communication is a major challenge due to gaps, inconsistencies and heterogeneity in practice.^23–25^ Despite its importance, communication of *C. auris* status at the time of interfacility transfer has not been standardized and most jurisdictions in the U.S. do not have such interfacility communication requirements.^25^ Among states mandating interfacility communication of multidrug-resistant organism status, compliance is heterogeneous, with <50% using standardized communication processes.^26^ Some strategies to overcome this barrier have been developed, such as statewide web-based registries (eg, Illinois XDRO registry^27^) for data exchange, but additional work is needed. Standardization and automation of clinical information sharing may improve the quality and safety of patient transitions, while simultaneously preventing *C. auris* spread at receiving facilities due to rapid initiation of prevention measures.

This study has several limitations. The survey was distributed to U.S.-based acute care hospitals, which may limit generalizability to global health systems. The SRN, as a consortium of hospitals with explicit interest in infection control practices, may not represent all acute care facilities in the U.S. and thus generalizability may be limited. Most participants self-identified as teaching hospitals; this may also limit generalizability because teaching hospitals may have more resources than community hospitals to implement prevention programs.^22^ As such, our findings may overestimate the true prevalence of some practices, such as *C. auris* screening. The survey instructed participating facilities to describe their own screening practices, but not those performed by the public health department. Facilities that were reliant on public health for surveillance would not have reported this practice, and this should be taken into consideration when interpreting results. Inherent limitations of survey-based research should also be considered, such as non-response, recall and social desirability biases.^28,29^ The influence of non-response bias is anticipated to be low within this report, as the majority of respondents completed all questions: four declined to answer detailed questions about screening for *C. auris*, but all respondents completed the sections detailing infection prevention practices. Strengths of this study include alignment of reported data with the previously described epidemiology of *C. auris*; for example, reports of antifungal-resistant strains are present in previously described geographic regions of the Northeastern U.S.^1–5^ This study reflects the experience of the SRN facilities at the time of survey completion in 2024, and Figure 2 should not be interpreted with the implication that resistant isolates of *C. auris* are limited to regions marked within the figure.

In this survey of U.S.-based acute care SRN hospitals, we identified multiple barriers and potential solutions to improve *C. auris* infection prevention and control. Increased education and support for effective environmental disinfection and interfacility communication are potential targets for future interventions. Additionally, development of effective *C. auris* decolonization regimens and more discrete guidance for surveillance programs should be prioritized.

## Supporting information

Lichota et al. supplementary material Lichota et al. supplementary material

## Supplementary material

The supplementary material for this article can be found at https://doi.org/10.1017/ice.2026.10396
