# Supplementary material for: Candidozyma auris prevention practices in the United States: insights from the SHEA Research Network
Source: Infect Control Hosp Epidemiol. 2026 Feb 2;47(4):373–8. doi: 10.1017/ice.2026.10396 (PMC12888079; doi:10.1017/ice.2026.10396)
Supplement: Lichota et al. supplementary material [file S0899823X26103961sup001.pdf]

Candida auris Prevention

You are being invited to participate in a research study that is being conducted by Dr. Sarah Sansom, DO, in the Department of Infectious Diseases at Rush University Medical Center. The purpose of this study is to examine experiences and policies to prevent transmission of Candida auris across healthcare facilities.

Taking part in this study is completely voluntary. You do not have to participate in this study and may choose to leave the study at any time. The person(s) answering this survey should be part of a US-based SRN Facility. Survey participants should be familiar with healthcare epidemiology workflows and policies within their institutions. This study will ask participants to describe their own role at their facility, with an emphasis on patient facing staff, as well as characteristics of patients at their facility. We will also query institutional screening protocols, laboratory capabilities, and current methods of surveillance and isolation for suspected cases of Candida auris.

If you agree to participate in this study, anticipate a survey completion time estimated at 20 minutes to complete. If incompletely filled out, this survey can be saved and returned to at a later date for completion. You will need to enter your institution's SRN ID to complete this survey.

Please note that no identifying information is collected in this study other than the institutional SRN ID. Information collected for this study may be used for future research. You may not directly benefit from taking part in this study, but we hope that knowledge gained from this study provide benefits across healthcare systems in the prevention of transmission Candida auris in the future .

Please add your SRN site ID number ______________________________

Part I. Facility and Staff Characteristics

Indicate your role at your institution; select all that apply.

- ☐ Hospital epidemiologist
- ☐ Associate hospital epidemiologist
- ☐ Infectious disease specialist
- ☐ Administrator
- ☐ Infection control practitioner
- ☐ Infection control manager
- ☐ Director of antimicrobial stewardship
- ☐ General Pharmacist
- ☐ Infectious Diseases or Stewardship Pharmacist
- ☐ Microbiologist
- ☐ Other

Please clarify institutional role.

____

What kind of facility is your primary place of work? Select all that apply.

- ☐ Teaching hospital
- ☐ Public/Government hospital
- ☐ Private hospital
- ☐ Veteran's Affairs (VA) hospital
- ☐ Rural hospital
- ☐ Community hospital
- ☐ Critical access hospital
- ☐ Tertiary care hospital
- ☐ Other

Please clarify facility type.

____

Please clarify practice location if primary employer is academia (ie, teaching hospital).

____

In which geographic region is your facility located?

- ○ Northwest United States
- ○ Midwest United States
- ○ Southeast United States
- ○ Southwest United States
- ○ Northeast United States
- ○ Outside of the United States

How many beds does your facility have?

- ○ less than 200 beds
- ○ 200-500 beds
- ○ greater than 500 beds

What is the typical staffing ratio for nurses at your facility for general medical level of care? Please estimate the number of patients cared for by one (1) nurse.

____
(• enter numerical value to indicate ratio : one nurse for _______ patients.)

What is the typical staffing ratio for nurses at your facility for intensive care unit level of care? Please estimate the number of patients cared for by one (1) nurse.

____
(• enter numerical value to indicate ratio : one nurse for _______ patients.)

What types of patient rooms are most common in your inpatient areas? Select all that apply.

- ☐ Single patient rooms
- ☐ Semi-private rooms (shared by 2 people)
- ☐ Shared rooms (shared by up to 4 people)
- ☐ Wards (shared by >4 people)

Which of the following staff members are involved in your infection prevention and control program? Select all that apply.	☐ Hospital epidemiologist<br>☐ Infectious Disease physician<br>☐ Other physician (please specify specialty below)<br>☐ Nurse, Infection control certified<br>☐ Nurse, not infection control certified<br>☐ Advanced Practice Providers (APPs) and/or Nurse Practitioners (NPs)<br>☐ Pharmacist<br>☐ Microbiologist<br>☐ Environmental services staff<br>☐ Employee/occupational health staff<br>☐ Hospital administrator<br>☐ Other
Please clarify physician specialty.	__________________________________
For "other" selected, please indicate below.	__________________________________
Infection control staff in your hospital are employed as (select all that apply) :	☐ Full time staff for infection control duties only<br>☐ Part time for staff infection control duties only<br>☐ Staff who have both infection control and other clinical/administrative responsibilities<br>☐ Other
Please clarify staff member employment status.	__________________________________

Part II. Facility Experiences with Candida auris

Has your facility ever identified any patient infected or colonized with Candida auris?	◯ Yes<br>◯ No
Approximately how many patients are admitted and/or identified with Candida auris at your facility each month?	◯ 0, we have not identified any patients with Candida auris<br>◯ 1 patient<br>◯ 2-5 patients<br>◯ 6-9 patients<br>◯ 10 or more patients
Has your facility ever experienced an outbreak (e.g., suspected transmission within your facility) of Candida auris?	◯ Yes<br>◯ No
Has your facility identified any Candida auris isolates that are resistant to 3 or more antifungal classes (i.e., azoles, echinocandin, and polyene/amphotericin)?	◯ Yes<br>◯ No

Part III. Candida auris Control Practices

Does your facility have a written policy on Candida auris prevention?	◯ Yes<br>◯ No
Does your facility perform screening for Candida auris?	◯ Yes<br>◯ No
Please select body sites used for screening at your facility (select all that apply):	☐ Axilla/armpit<br>☐ Inguinal crease/groin<br>☐ Anterior nares/nose<br>☐ Other (please specify):________________
Please clarify other body site used for screening.	______________________________
What screening approaches are used in your facility? (select all that apply)	◯ Admission Screening: patients are tested at the time that they are admitted to the facility<br>◯ Response-based Screening: this does not include point prevalence surveys conducted by public health.
Please further clarify how admission screening occurs at your facility.	◯ All patients admitted to the facility are screened<br>◯ All patients admitted to specific units in the facility are screened (clarify which units below)<br>◯ Targeted patients are screened (clarify targeted patients below)
Please clarify which specific units in the facility are screened upon admission.	☐ Intensive care unit patients are screened upon admission<br>☐ Specific non-ICU patients (e.g., transplant or oncology) are screened upon admission<br>☐ Other, please specify :
Please clarify which specific non- ICU units in the facility are screened upon admission.	______________________________
Please clarify any other units not described above which are screened upon admission.	______________________________
Please further clarify how targeted screening occurs at your facility.	◯ Only patients admitted from another healthcare facility are screened<br>◯ Only patients with a history of other multidrug-resistant organisms (e.g., CRE) are screened<br>◯ Only patients with invasive medical devices (e.g., tracheostomy) are screened<br>◯ Other (please specify): ________
Please clarify which other patient group is targeted for screening of C. auris at your facility.	______________________________
Please clarify the type of originating facility which is targeted for screening of C. auris at your facility.	◯ Acute care hospitals<br>◯ Long term acute care hospitals (LTACH)<br>◯ Ventilator-capable skilled nursing facilities (vSNF)<br>◯ Any skilled nursing facility<br>◯ Other (please specify)

Please explain other facility type.	__________________________
Please further clarify how response based screening occurs at your facility.	☐ Screening is performed for prevention (i.e., routinely performed to detect asymptomatic cases)<br>☐ Screening is performed in response to known C. auris cases<br>☐ Screening is performed for patients with an epidemiologic link to a Candida auris patient<br>☐ Screening is performed for point prevalence surveys initiated by the facility
What laboratory method does your facility use to screen for Candida auris? (select all that apply)	☐ Culture, performed in-house at your facility<br>☐ Culture, send out test<br>☐ PCR, performed in-house at your facility<br>☐ PCR, send out test<br>☐ Other (please specify): ________________
Average turnaround time from In-House Culture collection to result: _____ days	__________________________
Average turnaround time from Send_Out Culture collection to result: _____ days	__________________________
Average turnaround time from In House PCR collection to result: _____ days	__________________________
Average turnaround time from Send Out PCR collection to result: _____ days	__________________________
Please specify other laboratory method used by your facility for C. auris screening.	__________________________
What infection control and prevention measures does your facility use when a patient is suspected or identified as infected or colonized with Candida auris? (select all that apply)	☐ Isolation<br>☐ Disinfection<br>☐ Other Methods
Isolation measures at your facility include (select all that apply) :	☐ Pre-emptive isolation while awaiting screening result<br>☐ Contact isolation (i.e., gown and gloves)<br>☐ Single patient room<br>☐ Cohorting of patients (shared room or ward with other patients who have C. auris)<br>☐ Cohorting of healthcare staff to care for patients with C. auris<br>☐ Signage at patient room to notify staff of isolation status

Disinfection measures at your facility include (select all that apply) :	☐ Increased frequency of environmental disinfection of patient room<br>☐ Addition of other adjunctive environmental disinfection methods (e.g., UV disinfection, hydrogen peroxide mist).<br>☐ Ensure use of cleaning products with claims for C. auris disinfection (EPA List P

	bial-products-effective-against-candida-auris-list)<br>☐ Enhanced cleaning of shared patient equipment
Please clarify what other adjunctive environmental disinfection methods are used at your facility.	__________________________________
Other infection control measures taken at your facility include (select all that apply) :	☐ Antiseptic bathing<br>☐ Other (please specify):_____________________
Please specify which products are used specifically for antiseptic bathing against C. auris in your facility.	__________________________________
Please describe any other infection prevention measures taken at your facility.	__________________________________
Do other healthcare facilities in your area have a notification system to alert each other when patients with Candida auris are being transferred? Select all that apply.	☐ Yes, transfer documents provide this information<br>☐ Yes, verbal communication usually occurs<br>☐ Yes, a health information exchange-enabled electronic alert system provides this information<br>☐ Other (free text)<br>☐ No
Please clarify notification system type for your facility.	__________________________________
What additional measures are taken to prevent transmission of Candida auris while a colonized or infected patient is hospitalized? Select all that apply.	☐ None<br>☐ Signage to notify staff of isolation status<br>☐ Additional cleaning of patient's room<br>☐ Additional equipment cleaning<br>☐ Cohorting of patients<br>☐ Antiseptic bathing<br>☐ Cohorting of healthcare staff<br>☐ Other (free text)
Please describe additional measures to prevent transmission of C. auris at your facility.	__________________________________
What criteria does your facility use to determine the duration of isolation for patients harboring Candida auris?	☐ Isolate indefinitely<br>☐ Isolate only during the hospitalization that C. auris was identified, but not on subsequent admissions<br>☐ Isolate only while there is an active infection with C. auris<br>☐ Isolate until screening cultures are negative<br>☐ Other de-isolation protocol
Please clarify other de-isolation protocol used at your institution.	__________________________________

In your opinion, what are the most important barriers that should be addressed to prevent the spread of Candida auris in your facility? Rank the top 3 barriers, with 1 being the most important.

	First in importance	Second in importance	Third in importance
Lack of training for frontline staff	○	○	○
Lack of microbiologic and/or diagnostic services	○	○	○
Lack of access to infection data	○	○	○
Lack of access to information on infection control	○	○	○
Lack of communication between healthcare facilities	○	○	○
Lack of infection control at outside healthcare facilities	○	○	○
Lack of administrative support	○	○	○
Lack of supplies	○	○	○
Lack of staffing	○	○	○
Space constraints (inability to isolate in single room)	○	○	○
Lack of electronic health record integration across facilities	○	○	○
Other (please specify)	○	○	○

If Other barrier is ranked above, please clarify.

____

In your opinion, what are the most important facilitating factors that help prevent the spread of Candida auris in your facility? Rank the top 3 facilitating factors, with 1 being the most important.

	First in importance	Second in importance	Third in importance
Training for frontline staff	◯	◯	◯
Microbiologic and/or diagnostic services	◯	◯	◯
Access to infection data	◯	◯	◯
Access to information on infection control	◯	◯	◯
Communication between healthcare facilities	◯	◯	◯
Infection control at outside healthcare facilities	◯	◯	◯
Administrative support	◯	◯	◯
Adequate supplies	◯	◯	◯
Adequate staffing	◯	◯	◯
Ability to isolate a patient in a single room	◯	◯	◯
Electronic health record integration across facilities	◯	◯	◯
Other (please specify)	◯	◯	◯

If Other factor is ranked above, please specify. ______________________________

In your opinion, development of which of the following tools would be most helpful to support your ability to respond to Candida auris in your facility? Rank the top 3 tools, with 1 being the most important.

	First in importance	Second in importance	Third in importance
Standardized protocols for C. auris screening	○	○	○
Standardized protocols for C. auris isolation	○	○	○
Standardized protocols for C. auris treatment	○	○	○
Effective patient decolonization regimen for C. auris	○	○	○
New or improved environmental disinfection protocols or tools	○	○	○
Improved communication at time of patient transfer between facilities	○	○	○
Improved training resources for frontline staff	○	○	○
Improved training resources for infection prevention staff	○	○	○
Improved access to C. auris screening tests or test results	○	○	○
Faster turnaround time for C. auris screening tests	○	○	○
Other (please specify)	○	○	○

If Other tool is ranked above, please specify. ______________________
